# Effects of Attachment of Plastic Aligner in Closing of Diastema of Maxillary Dentition by Finite Element Method

**DOI:** 10.1155/2019/1075097

**Published:** 2019-03-03

**Authors:** Yukiko Yokoi, Atsushi Arai, Jun Kawamura, Tomoko Uozumi, Yohei Usui, Norimasa Okafuji

**Affiliations:** ^1^Department of Pediatric Dentistry, Matsumoto Dental University School of Dentistry, Matsumoto, Japan; ^2^Department of Orthodontics, Matsumoto Dental University School of Dentistry, Matsumoto, Japan; ^3^Department of Dental Materials Science, School of Dentistry, Aichi-Gakuin University, Nagoya, Japan; ^4^Department of Oral Health Promotion, Matsumoto Dental University School of Dentistry, Matsumoto, Japan

## Abstract

The aim of this study was to clarify the effect of attachment on tooth movement produced by a plastic aligner. Closing of a diastema, in which the maxillary right and left central incisors moved bodily, was simulated using a finite element method. Long-term orthodontic movements of the maxillary dentition were simulated by accumulating the initial displacement of teeth produced by elastic deformation of the periodontal ligament. The incisor tipped and rotated just after placement of the aligner irrespective of the attachment. After a sufficiently long time, the incisor was upright and moved bodily in the aligner with attachment, but the incisor remained tipped in the aligner without attachment. It was demonstrated that the attachment was effective for achieving bodily movement.

## 1. Introduction

Transparent aligner treatment has an esthetic advantage over conventional wire-bracket treatment. In addition to the esthetic standpoint, it has a very simple principle, in which an aligner made based on a target or predicted dentition is only placed on the patient's dentition. In recent aligners, attachments have been used for active control of tooth movement. Aligner treatment is easier than wire-bracket orthodontics, in which an appropriate appliance design is necessary to control the tooth movement.

Clinical accuracy of aligner treatment has been measured by comparing the virtual model of the target dentition with the achieved dentition after treatment. Kravitz et al. evaluated the accuracies of expansion, constriction, intrusion, extrusion, mesiodistal tip, labiolingual tip, and rotation of the anterior teeth and reported a mean accuracy of 41% [1]. In their study, attachment had no effect on the accuracy of canine rotation [2]. Krieger et al. reported that tooth corrections in the vertical plane were difficult to realize, but frontal crowding was successfully corrected [3]. In a recent clinical study by Simon et al., the accuracies for incisor torque, premolar derotation, and molar distalization were 42%, 40%, and 87%, respectively [4]. Also, bodily tooth movements could be accomplished. Various types of tooth movement seemed to be achieved successfully by aligner treatments.

In addition to the clinical studies, a few *in vitro* studies have been carried out to evaluate forces and moments applied from an aligner to the tooth [5, 6]. They showed that forces and moments generated by aligners were within the range of usual orthodontic forces. However, it is not clear how aligners transmit forces and moments to teeth and how teeth were moved by aligners [7].

The purpose of this study is to investigate the mechanics of tooth movement produced by an aligner, especially the effect of attachment on the bodily movement. For this purpose, the closing of a diastema, in which the central incisors move bodily with an aligner, was simulated using a finite element method (FEM).

## 2. Materials and Methods

### 2.1. Finite Element Model

In usual aligner treatments, many aligners are replaced sequentially until the teeth move to their target positions. Teeth are moved about 0.2 mm in each aligner. In the present study, we simulated a tooth movement that is produced by one aligner.

**Figure 1:**
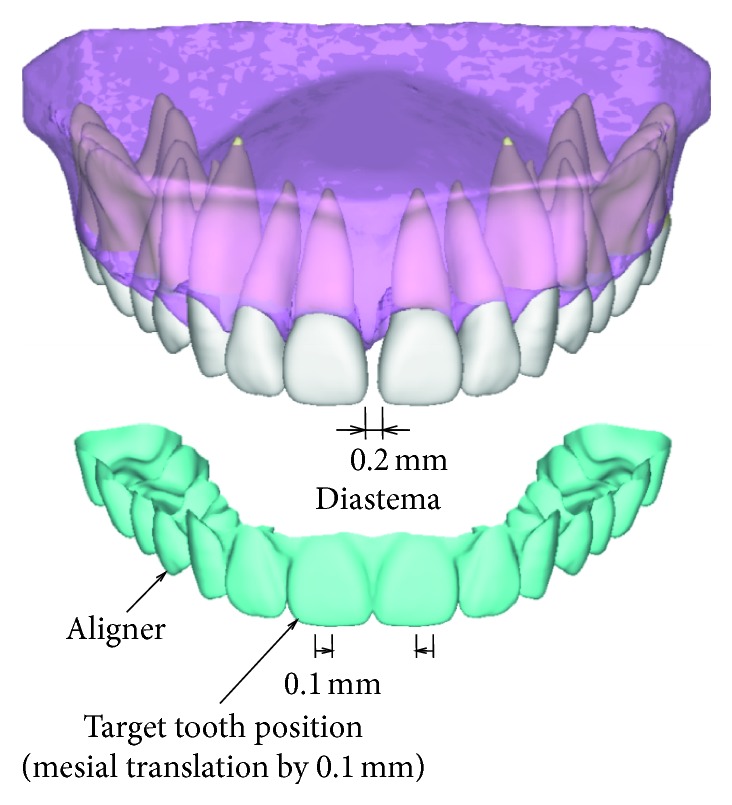
Closing of diastema with a plastic aligner.

**Figure 2:**
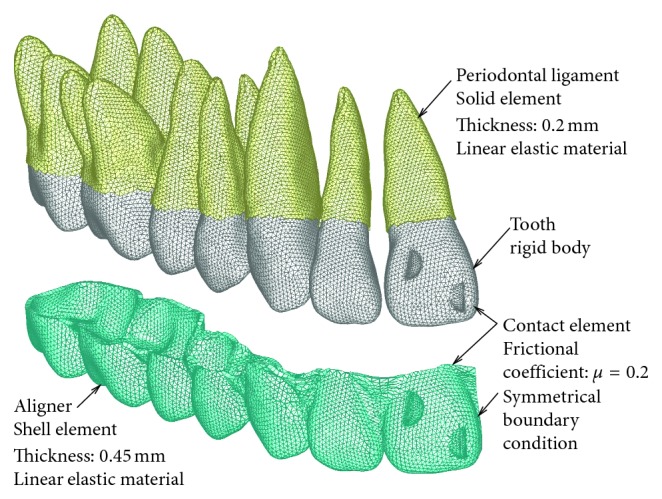
Finite element model of the aligner and dentition.

To close a diastema of 0.2 mm in the maxillary dentition, an aligner was used to translate right and left central incisors mesially by 0.1 mm (Figure 1). Assuming the upper dentition to be bilaterally symmetrical, finite element models of teeth and an aligner were constructed only for the right side (Figure 2).

Finite element models of the teeth were made based on a dental study model (i21D-400C, Nissin Dental Products Inc., Kyoto, Japan) [8]. They were made by the following three steps. First, sequential sectional images of the dental study model were taken using dental cone beam computed tomography (CBCT), AZ300CT (Asahi Roentgen, Co., Ltd., Kyoto, Japan). Second, using the 3D-modeling software, 3D-Doctor (Able Software Corp., Lexington, Massachusetts, USA), a stereolithographic (STL) model of each tooth, was constructed. Third, the surface of the STL model was divided with 3-node triangular shell elements using meshing software, ANSYS AI∗Environment (ANSYS Japan, Tokyo, Japan). Periodontal ligament (PDL) with uniform thickness of 0.2 mm was constructed on the root with 6-node triangular prismatic solid elements. Total numbers of element and node of the teeth with the PDL were 67210 and 33860, respectively. The element size was about 0.5 mm.

Using the STL models of tooth crowns, a finite element model of an aligner was constructed manually as follows. Arranging the crown models in the target positions without diastema, their approximal surfaces were merged and formed like an aligner by using a polygon editor, Metasequoia 4 (Tetraface Inc. Tokyo, Japan). The aligner was divided with 3-node triangular shell elements of 0.45 mm thickness. The number of element and node of the aligner were 16144 and 8230, respectively. The element size was about 0.5 mm. This aligner model was fitted into the target dentition without any clearance gap.

Young's modulus and Poisson's ratio of the aligner are shown in Table 1, which are typical of thermoplastic materials. To examine the effect of elastic deformation of the aligner, its Young's modulus was increased by up to 1000 times, to 2000 GPa. In this case, elastic deformation of the aligner was eliminated. Attachment was constructed both on the incisor and the aligner by referring to the Invisalign system [9]. A symmetrical boundary condition was applied to the midsection of the aligner.

The periodontal ligament (PDL) was assumed to be a linear elastic film of 0.2 mm thickness, and its Young's modulus and Poisson's ratio are shown in Table 1, respectively [10]. When using these elastic moduli, the mobilities of the upper first premolar calculated by the finite element method became about the same as those measured *in vivo* [11].

The teeth and the alveolar bone were assumed to be rigid bodies because their Young's moduli (about 15 GPa) were very much larger than those of both the PDL and the aligner. Under this assumption, nodes on the alveolar socket, namely, nodes on the outer surface of the PDL, were defined as a rigid surface and were moved together with orthodontic tooth movement. A finite element model of the alveolar bone was therefore unnecessary.

Contact elements were set between the aligner and the crown of tooth. The crown was in contact with the neutral surface of the aligner. The frictional coefficients between them were assumed to be 0.2.

In actual treatment, to close a diastema, an aligner made based on the target dentition without diastema is placed on the patient's dentition with the diastema. Such placement could not be simulated by the FEM. Instead, each tooth was put in the aligner, and then a 0.2 mm diastema was made by translating the alveolar socket of the central incisor distally by 0.1 mm. This activation process was equivalent to the actual placement of the aligner on the dentition.

### 2.2. Simulation Method for Orthodontic Tooth Movement

**Figure 3:**
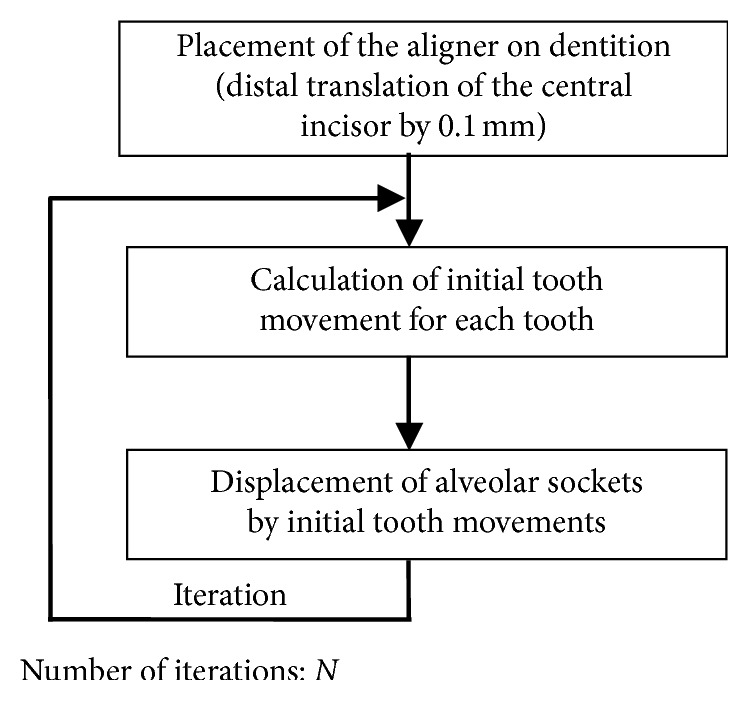
Calculation procedure for simulating orthodontic tooth movement. *N* is equivalent to time elapsed.

In orthodontic tooth movement, the alveolar socket moves due to remodeling of the alveolar bone. This process was simulated by the FEM. The outer surface of the PDL, which coincides with the alveolar socket, was moved based on the initial movement produced by elastic deformation of the PDL. The simulation procedure is shown in Figure 3, which is similar to that in the previous article [10]. First, the aligner was placed on the dentition, that is, the alveolar socket of the central incisor was translated distally by 0.1 mm. Second, the initial movement of each tooth was calculated. The teeth moved as rigid bodies. Third, the alveolar socket of each tooth was moved by the initial movement, that is, displacement and rotation of the initial movement were applied to the node on the outer surface of the PDL. By repeating the second and third steps, the teeth moved step by step. The force system acting on the teeth was updated at each step. The number of the steps, *N*, corresponds to the time elapsed after placement of the aligner. But, *N* could not be converted to an actual time. The repeating calculation was executed automatically using a programing language APDL in the FEM software, ANSYS 11.0 (ANSYS Japan, Tokyo, Japan).

## 3. Results

**Figure 4:**
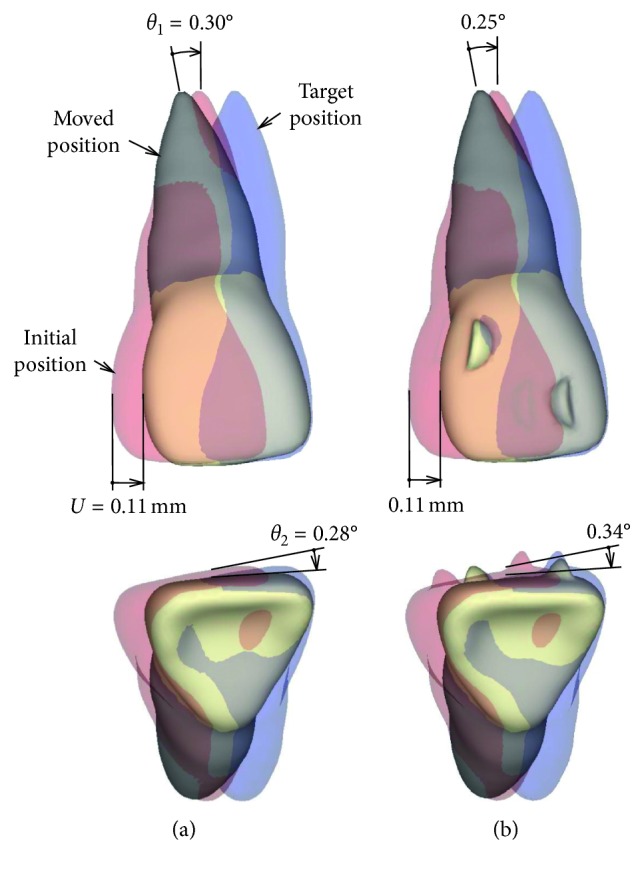
Initial movement just after placement of the aligner: (a) without attachment; (b) with attachment. Movement is magnified 20 times.

Figure 4 shows movement of the central incisor just after placement of the aligner. This movement was produced by elastic deformation of the PDL and the aligner. The profile of the moved incisor was depicted by magnifying its displacement 20 times. The red profile indicates the initial position, and the blue profile indicates the target position at which the incisor translates mesially by 0.1 mm. In Figure 4, the central incisor tipped and rotated irrespective of the attachment. When increasing Young's modulus of the aligner up to 2000 GPa, the incisor translated completely to the target position irrespective of the attachment.

**Figure 5:**
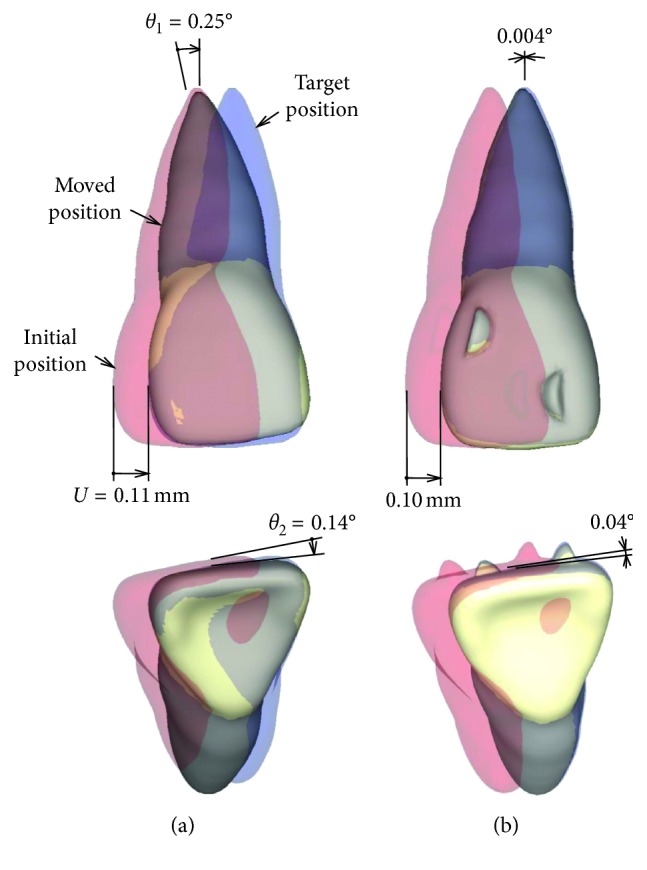
Movement pattern after a sufficiently long time, *N*=500: (a) without attachment; (b) with attachment. Movement is magnified 20 times.

Figure 5 shows the moved position of the incisor at the number of iterations *N*=500. In the aligner without attachment, the crown of the incisor moved to the target position, but the root apex hardly moved (Figure 5(a)). In the aligner with the attachment, the incisor overlapped completely on the target position (blue tooth), meaning the efficacy of movement was almost 100% (Figure 5(b)). In this case, the incisor could be moved bodily and the other teeth hardly moved.

**Figure 6:**
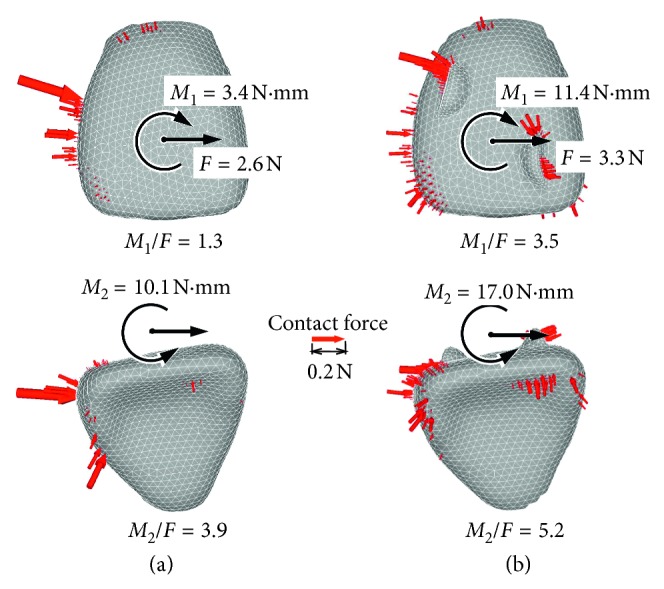
Force system and contact force just after placement of the aligner: (a) without attachment; (b) with attachment.

Figure 6 shows contact forces acting on the crown just after placement of the aligner. In the aligner without attachment, the contact forces acted only on the distal side of the crown (Figure 6(a)). These contact forces resulted in a mesial force *F* and moments *M*_1_ and *M*_2_ at the bracket position. In the aligner with attachment, the contact forces acted not only on the distal side of the crown but also on the attachments, thereby increasing force and moments at the bracket position (Figure 6(b)). The moment *M*_1_ was increased to almost 3 times by the attachment.

**Figure 7:**
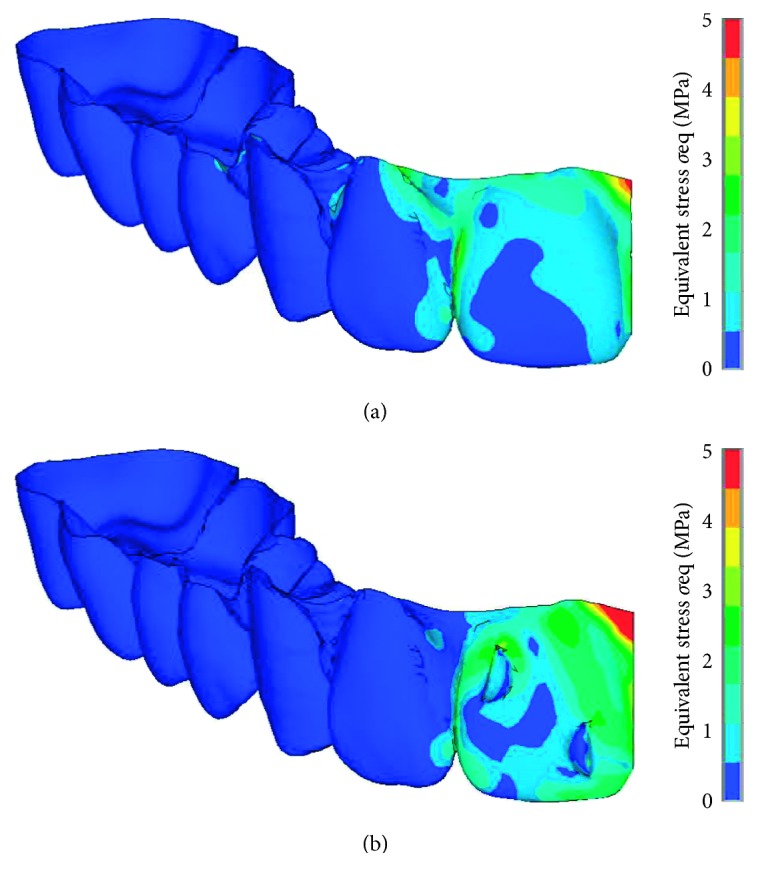
Equivalent stress in the aligner just after placement: (a) without attachment; (b) with attachment.

Figure 7 shows the equivalent stress in the aligner just after placement of the aligner. The stress was produced only in the incisor region of the aligner and was concentrated at the midsection.

## 4. Discussion

### 4.1. Effect of the Attachment

The attachment hardly affected movement of the incisor just after the placement of the aligner (Figure 4(b)). The reason for this can be explained as follows. The attachment increased the moment-to-force ratio *M*_1_*/F* for preventing tipping (Figure 6(b)). But, its magnitude (*M*_1_*/F* = 3.5) was less than that required for bodily movement, and thereby the incisor tipped (Figure 4(b)). Although the crown moved to the target position, the root apex moved distally. By the same reasoning, the incisor rotated slightly about the vertical axis. The tipping and rotation of the incisor were due to elastic deformation of the aligner. When Young's modulus of the aligner was increased up to 1000 times to eliminate the elastic deformation, the incisor moved bodily to the target position.

As mentioned above, the attachment had no effect on the initial movement (Figure 4). However, this result was different from those obtained by the previous FEM studies [12, 13]. In these studies, the initial movements were calculated in the case where the canine was moved distally using aligners with and without attachment. In each result, the canine tipped without attachment but moved bodily with an attachment, namely, the attachment was effective for bodily movement. The kind of the tooth, shape of the aligner and attachment, and mechanical properties of the PDL were different between the previous and the present studies. Although we investigated their studies carefully, it was impossible to explain the cause of difference between the results of the previous and the present studies.

The effect of the attachment became clear after allowing sufficient time (*N*=500). In the aligner with attachment, tipping and rotation angles of the incisor became almost 0, that is, the incisor moved bodily (Figure 5(b)). In the aligner without attachment, the incisor remained in almost the same position as during the initial movement (Figure 5(a)).

Mechanics of the attachment can be explained as follows. The incisor tipped and rotated at the placement of the aligner due to an elastic deformation of the aligner. As the alveolar socket of the incisor moved distally, the distal force acting on the incisor was decreased so that the moment-to-force ratio was increased. The incisor began to be upright and moved bodily after a sufficient elapsed time (Figure 5(b)). During movement of the incisor, the aligner behaved as a spring, and the exerted force was transmitted to the incisor through the attachment (Figure 6(b)). It seemed that the attachment of the aligner gripped the incisor. The incisor tipped at first and then to be upright. Such a movement pattern was the same as that produced by a retraction spring [10].

When closing the diastema, because right and left incisors moved mesially each other, forces acting on both incisors were balanced and hardly affected the other teeth. The elastic deformation was produced mainly in the region of the central incisor (Figure 7). Stress concentrations occurred near the attachment and at the midsection of the aligner. These regions will become weak points in strength of the aligner.

### 4.2. Finite Element Method

In the present simulation, effects of the attachment could be examined under the same property of tooth movement. This is an advantage of the finite element simulation over randomized controlled trials, where the effect of the attachment may be buried under the individual difference in tooth movement property. Although such a finite element simulation can never take the place of randomized trials, it is helpful to understand how and why a mechanical factor affects tooth movement.

The finite element model of the aligner in the present simulations could be placed on the target dentition without any clearance gap. It was an ideal sort of aligner. The tooth movements simulated in the present study will also be ideal tooth movements. There must be some clearance gaps between the aligner and dentition in clinical conditions. However, it was difficult to include the clearance gap in the simulation model, because the amount and distribution of clearance gap between the aligner and dentition has not been clarified.

In the present study, not only the initial movement but also the orthodontic tooth movement was simulated. The initial movement is produced by an elastic deformation of the PDL. And, the orthodontic tooth movement is a biological displacement of the alveolar socket due to remodeling of the alveolar bone. Although mechanisms of both movements are different from each other, their movement patterns are very similar [14]. Hence, it was assumed that the orthodontic movement occurs in the same direction as in the initial movement.

## 5. Conclusion

The incisor tipped and rotated irrespective of the attachment just after placement of the aligner. After a sufficiently long time, the incisor was upright and moved bodily in the aligner with attachment, although the incisor remained tip in the aligner without attachment. The effectiveness of the attachment was demonstrated.

## Figures and Tables

**Table 1:** Mechanical properties of the aligner and the periodontal ligament (PDL).

	Young's modulus (MPa)	Poisson's ratio
Aligner	2000	0.4
PDL	0.13	0.45

## Data Availability

The data used to support the findings of this study are included within the article.
